# Early cardiac involvement in patients with hyperthyroidism evaluated by cardiac magnetic resonance feature tracking

**DOI:** 10.3389/fendo.2025.1566029

**Published:** 2025-06-02

**Authors:** Yi Zhang, Yiming Jia, Renbin Ge, Qian Luo, Xiance Zhao, Xing Xing, Fang Liu, Lei Zhang

**Affiliations:** ^1^ Department of Radiology, Shanghai General Hospital, Shanghai Jiao Tong University School of Medicine, Shanghai, China; ^2^ Department of Clinical Medical College, Anhui Medical University, Hefei, China; ^3^ Department of Clinical & Technical Support, Philips Healthcare, Shanghai, China; ^4^ Department of Cardiology, Shanghai General Hospital, Shanghai Jiao Tong University School of Medicine, Shanghai, China; ^5^ Department of Endocrinology and Metabolism, Shanghai General Hospital, Shanghai Jiao Tong University School of Medicine, Shanghai, China

**Keywords:** hyperthyroidism, atrial fibrillation, quantitative evaluation, feature tracking, cardiovascular resonance magnetic imaging

## Abstract

**Background:**

This study aimed to investigate the feasibility of quantitative assessment of myocardial injury in the early stages of the disease by myocardial strain in patients with hyperthyroidism.

**Methods and results:**

A total of 45 individuals in the hyperthyroid state with at least one index of cardiac involvement (e.g., cardiac symptoms, abnormal electrocardiogram, or abnormal cardiac biomarkers) and 26 healthy controls were subjected to cardiovascular magnetic resonance (CMR) to assess the left ventricular (LV) volumetry, LV mass index (LVMi), LV ejection fraction (LVEF), myocardial strain, and the total (TEF), passive (PEF), and active (AEF) left atrial (LA) emptying fractions. Patients with hyperthyroidism were classified into two categories according to the presence (*n* = 5) or absence (*n* = 40) of atrial fibrillation (AF). The LA reservoir, conduit, and booster strains were significantly impaired in all patients with hyperthyroidism (all *p* < 0.01). In contrast, the LA and LV ejection fractions, LVMi, and LV myocardial strain showed no significant differences between the cases without AF and the healthy controls (all *p* > 0.05). The LA-AEF and LVEF were significantly decreased in the hyperthyroidism cases with AF compared with the hyperthyroidism cases without AF, although no significant differences in the LA-TEF, PEF, and LVMi were detected. The receiver operating characteristic (ROC) curve analysis revealed superior diagnostic value for the LA reservoir strain, which distinguished hyperthyroidism cases from normal subjects with a sensitivity of 80.8% and a specificity of 80%.

**Conclusions:**

The LA strain reliably differentiates early cardiac involvement between patients with hyperthyroidism and healthy controls, whereas volumetric parameters and ventricular myocardial strain fail to do so.

## Introduction

Hyperthyroidism is a syndrome resulting from an excess of circulating free thyroxine caused by thyroid gland overactivity. Excessive thyroid hormone affects the energy production in normal myocytes, the intracellular metabolism, and the myofibril contraction (1), which enhances susceptibility to apoptosis and decreases the contractility of cardiomyocytes, ultimately resulting in cardiac dysfunction and heart failure (2, 3).

Hyperthyroidism was also reported to be associated with atrial fibrillation (AF) (4). In a recent population-based cohort study examining 586,460 patients with a median follow-up of 5.5 years, the highest relative risk of AF was detected in hyperthyroidism with suppressed thyroid-stimulating hormone (TSH) levels (5). In previous studies, excessive thyroid hormone increased the sympathetic function with decreasing heart rate variability and atrial refractory period by altering the β1-adrenergic and M2 muscarinic receptors of the heart in hyperthyroidism with AF (5, 6).

Numerous clinical studies focused on the LV function status in hyperthyroidism by applying echocardiography (7, 8). However, the standard parameters of ventricular global function yielded by echocardiography cannot detect subtle and regional ventricular functional alterations in the early phase of hyperthyroidism involving the heart. In addition, myocardial stain has recently attracted increasing attention in patients with hyperthyroidism (9).

Myocardial deformation imaging can help detect early systolic and diastolic dysfunction in multiple cardiovascular diseases (10–13). Myocardial strain, defined as the relative change in fiber length from the end-diastole to the end-systole, can be measured by speckle tracking echocardiography (STE) and cardiovascular magnetic resonance feature tracking (CMR-FT) (10–13). However, STE is highly user-dependent and images have lower signal-to-noise ratios than CMR scans (14). Analyzing the atrial and ventricular functions using volumetric and deformation parameters may provide insights into the early stages of cardiac involvement in patients with hyperthyroidism.

The present study enrolled patients with confirmed hyperthyroidism and suspected myocardial injury and assessed the left atrial (LA) and left ventricular (LV) functions and related deformation parameters by CMR to evaluate the feasibility of quantitative assessment of myocardial injury in the early stages of the disease by myocardial strain.

## Methods

### Study design and participants

A total of 45 patients diagnosed with hyperthyroidism were enrolled between January 2021 and January 2023. Patients were referred for cardiac MRI for suspected myocardial involvement based on cardiac symptoms, abnormal electrocardiography (ECG) findings, or abnormal cardiac biomarkers. All patients with hyperthyroidism had either cardiac symptoms (e.g., palpitation or dyspnea on exertion), abnormal ECG, or any abnormal cardiac biomarker [e.g., increased creatine kinase–myocardial band (CK-MB), high-sensitivity troponin I (hs-TnI), or type B natriuretic peptide (BNP)]. The exclusion criteria were: a) a history of heart disease such as coronary artery disease, congenital heart disease, cardiomyopathy, and valvular heart disease; b) diabetes mellitus with fasting blood glucose levels >6.1 mmol/L; and c) preexisting hypertension with blood pressure ≥140/90 mmHg.

A total of 26 healthy subjects (control group) were submitted to 3.0-T CMR imaging during the same period, who were matched to the above patients by age, sex, and body mass index (BMI) from the same community for another research.

This is a single-center retrospective, descriptive study. All procedures were approved by the Institutional Ethics Review Board of Shanghai General Hospital (no. 2022KY034). Signed informed consent was waived due to the retrospective nature of the study.

### Anthropometric and biochemical indices

The sex, age, height, body weight, heart rate, and blood pressure were obtained from patients’ hand-written charts and electronic medical records. Blood samples were collected within 3 days before MRI examination. The biochemical indices, including the levels of serum free triiodothyronine (FT3), total triiodothyronine (TT3), free tetraiodothyronine (FT4), total tetraiodothyronine (TT4), TSH, hs-TnI, CK-MB, and BNP, of patients with hyperthyroidism were assessed.

### Image acquisition

For patients with a high heart rate, β-blockers were used to control the heart rate before CMR examination. All participants underwent CMR on a 3.0-T MR scanner (Ingenia, Philips Healthcare, Best, the Netherlands). Cine images were obtained with a balanced steady-state free procession (b-SSFP) sequence in the LV long-axis (two, three, and four chambers) and short-axis (from the apex to the basal segment) planes. The imaging parameters were: slice thickness, 8 mm; repetition time (TR), 2.8–3.2 ms; echo time (TE), 1.4–1.5 ms; flip angle, 45°; matrix size, 160 × 138 to 176 × 192; field of view, 300 × 300 to 350 × 350 mm^2^; and temporal resolution, 15–25 ms, depending on the heart rate.

### Image analysis and post-processing

For post-processing, a dedicated cardiovascular software (cvi42, V5.11, Circle, Calgary, Canada) was used by a blinded experienced observer to analyze the structure, function, and myocardial strain of the left ventricle and the atrium in a random order. Volumetric analysis of the left ventricle and atrial time–volume curves were semi-automatically performed using the Simpson’s method and the biplane area length calculation technique, respectively (15). The LV cavity included the trabecular and papillary muscles. Subsequently, the left ventricular end-diastolic volume (LVEDV), end-systolic volume (LVESV), stroke volume (LVSV), ejection fraction (LVEF), and mass (LVM) were measured. All of the above measurements were normalized to body surface area (BSA). The maximum LA volume (LA-*V*_max_), the LA volume at the ventricular diastole prior to atrial contraction (LA-*V*_pac_), and the minimum LA volume (LA-*V*_min_) were derived from the time–volume curves of the left atrium according to previous reports (16–19). The total (TEF, corresponding to the atrial reservoir and atrial global functions), passive (PEF, corresponding to the atrial conduit function), and active (AEF, corresponding to atrial contractile booster pump function) LA emptying fractions were defined as follows (16–19):


$$\text{TEF}=\frac{\left(V\text{max}-V\text{min}\right)\times 100\%}{V\text{max}}$$

$$\text{PEF}=\frac{\left(V\text{max}-V\text{pac}\right)\times 100\%}{V\text{max}}$$

$$\text{AEF}=\frac{\left(V\text{pac}-V\text{min}\right)\times 100\%}{V\text{pac}}$$

**Figure 1:**
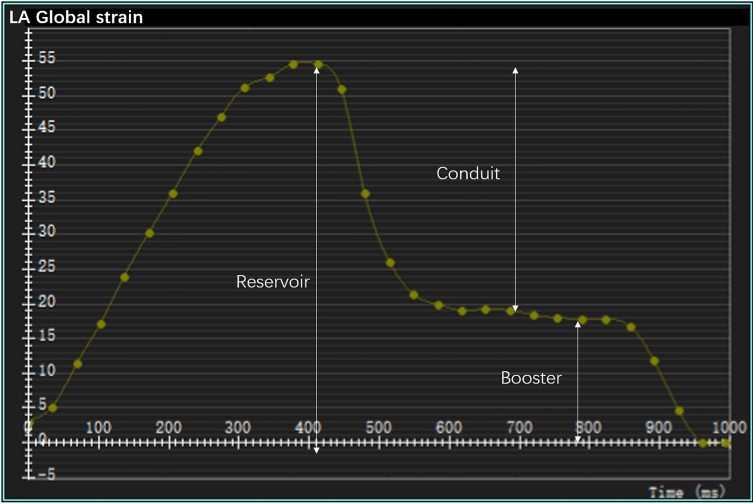
A representative left atrial strain curve with reservoir, conduit, and booster strains.

To calculate the LV strain, the endocardial and epicardial contours of the left ventricle were manually delineated on the short- and long-axis (two-, three-, and four-chamber views) cine images at the end diastole. Subsequently, the software automatically tracked the myocardial motions throughout the entire cardiac cycle. Finally, the global longitudinal strain (GLS), circumferential strain (GCS), and radial strain (GRS) of the left ventricle were evaluated. To determine the LA strain, the endocardial and epicardial borders of the left atrium were manually delineated at the end diastole in the two- and four-chamber views (19). In the analysis, the atrial appendage was included and the pulmonary veins excluded, as described previously (19–21). Finally, the software calculated the longitudinal LA strain automatically by tracking the contours throughout the whole cardiac cycle. The obtained strain curves were analyzed to identify peaks, utilizing the LV end-diastolic frame as a zero reference. Three aspects of the LA strain were analyzed: the reservoir strain (passive LA filling due to inflow from the pulmonary veins), the conduit strain (passive flow through the LA following mitral valve opening in the early LV filling phase), and the booster strain (active LA contraction during the late LV filling phase) (18, 22) (**Figure 1**).

### Reproducibility

We randomly selected 15 participants from the population. In these participants, the LV and LA measurements by myocardial strain were repeatedly performed by the same observer and another blinded observer.

### Statistical analysis

SPSS 20 (IBM Corp., Armonk, NY, USA) and MedCalc 20.015 were employed for data analysis. Continuous data were assessed for normality using the Kolmogorov–Smirnov test and by qualitative inspection of the *Q*–*Q* plots. Continuous data with normal distribution were expressed as the mean ± standard deviation and were compared using independent samples *t*-test (group pairs) or one-way analysis of variance (ANOVA; three groups). A one-way ANOVA was used for repeated measures with Bonferroni *post-hoc* adjustment. Continuous data with a skewed distribution were expressed as median (interquartile range, IQR) and were compared using the Kruskal–Wallis test. Categorical data were expressed as number (percentage) and were compared using the chi-square tests or Fisher’s exact test. The predictive ability of the various parameters was determined with receiver operating characteristic (ROC) curve analysis. DeLong’s test was performed to compare the area under the curve (AUC) values. The correlation between the LA emptying fractions and LA strain was examined using Spearman’s correlation analysis. A two-tailed *p* < 0.05 was considered statistically significant.

## Results

### Clinical characteristics of the study population

In total, 71 subjects were enrolled. **Table 1** shows the baseline clinical data of the 45 patients with hyperthyroidism [age = 45 years (33–54 years), 17 men, BMI = 22.1 ± 2.9 kg/m^2^] and 26 healthy controls [age = 36 years (29–48 years), 10 men, BMI = 25.6 ± 2.6 kg/m^2^]. The Hs-TnI and CK-MB were within the normal range in all patients. The BNP values in 17 (38%) of the 45 patients with hyperthyroidism were above the normal range. In total, 5 (11%) patients with hyperthyroidism had paroxysmal AF as indicated by ECG. There were 33 (73%) patients with hyperthyroidism who exhibited palpitation and 6 (13%) who manifested dyspnea on exertion. The median hyperthyroidism course was 4 months (IQR = 2–12 months). The sex, age, blood pressure, and BMI were comparable between the hyperthyroidism patients and healthy controls. Patients with hyperthyroidism had higher heart rates compared with the healthy controls [89 (80–98) *vs*. 65 (61–70), *p* < 0.001].

**Table 1:** Clinical characteristics of the study population.

Variable	Hyperthyroidism (*n* = 45)	Control (*n* = 26)	*p*-value
Age (years)	45 (33–54)	36 (29–48)	0.088
Men, *n* (%)	17 (38)	10 (38)	0.954
BMI (kg/m^2^)	22.1 ± 2.9	25.6 ± 2.6	0.461
Hyperthyroidism duration (months)	4 (2–12)		
HR (bpm)	89 (80–98)	65 (61–70)	<0.001
DBP (mmHg)	78 (75–82)	76 (74–80)	0.270
SBP (mmHg)	124 (120–126)	124 (120–127)	0.702
Symptoms, *n* (%)			
Palpitations	33 (73)	–	–
Dyspnea on exertion	6 (13)	–	–
Atrial fibrillation	5 (11)	–	–
Medication use, *n* (%)			
Antithyroid medication	29 (64)	–	–
β-blockers	36 (80)	–	–
FT3 (pmol/L)	12.3 (6.2–20.6)		
TT3 (nmol/L)	4.2 (2.2–5.3)		
FT4 (pmol/L)	40.3 (16.6–66.1)		
TT4(nmol/L)	199.3 ± 81.6		
TSH (μIU/ml)	0.07 (0.05–0.14)		
BNP (pg/ml)	79.4 (30.4–147.7)		
CK-MB (ng/ml)	0.83 (0.58–1.2)		
hs-TnI (ng/ml)	0 (0–0.01)		

Data are the mean ± SD, percentage (number of participants), or median (interquartile range), as appropriate.

*BMI*, body mass index; *HR*, heart rate; *SBP*, systolic blood pressure; *DBP*, diastolic blood pressure; *BNP*, type B natriuretic; *FT3*, serum free triiodothyronine; *TT3*, total triiodothyronine; *FT4*, free tetraiodothyronine; *TT4*, total tetraiodothyronine; *TSH*, thyroid-stimulating hormone; *hs-TnI*, high-sensitivity troponin I; *CK-MB*, creatine kinase–myocardial band.

Previous studies have indicated that patients with AF exhibit decreased LA myocardial strain (9). This study aimed to investigate whether myocardial strain is altered in hyperthyroidism without AF. Patients with hyperthyroidism were divided into two groups: those with AF (*n* = 5, 11%) and those without AF (*n* = 40, 89%). No significant differences were observed in the age, sex, left ventricular mass index (LVMi), LA total emptying fraction (LA-TEF), and LA peak emptying fraction (LA-PEF) among the AF, non-AF, and control groups. Furthermore, the AF group showed no significant differences in the hyperthyroidism duration or the laboratory parameters (FT3, TT3, FT4, TT4, TSH, and BNP) when compared with the non-AF group.

### Comparisons between the non-AF and healthy control groups

Compared with the healthy controls, the hyperthyroidism patients without AF showed a larger left ventricle (LVEDV index, 79.0 ± 14.5 *vs*. 69.6 ± 10.7 ml/m^2^, *p* = 0.006) and a higher LVSV index (48.6 ± 8.6 *vs*. 42.1 ± 6.8 ml/m^2^, *p* = 0.002) (**Table 2**). The LA emptying fractions and the LV myocardial strain showed no significant differences between the healthy controls and the hyperthyroidism patients without AF.

**Table 2:** Baseline and MRI characteristics of the study population.

Variable	AF (*n* = 5)	Non-AF (*n* = 40)	Control (*n* = 26)	*p*-value
Age (years)	52 (39–57)	42 (32–54)	36 (29–48)	0.117
Men, *n* (%)	3 (60)	14 (35)	10 (38)	0.554
Hyperthyroidism duration (months)	12 (4–18)	4 (1–12)	–	0.264
LVEDV index (ml/m^2^)	91.0 ± 9.8**	79.0 ± 14.5**	69.6 ± 10.7	0.001
LVESV index (ml/m^2^)	41.4 ± 6.6*,**	30.6 ± 7.7	27.7 ± 5.1	<0.001*
LVSV index (ml/m^2^)	49.6 ± 7.4	48.6 ± 8.6**	42.1 ± 6.8	0.005
LVEF (%)	56.2 ± 5.0*,**	62.0 ± 3.9	60.4 ± 3.4	0.007
LVM index (g/m^2^)	49.6 (41.7–52.9)	41.6 (39.4–47.0)	42.9 (36.4–48.2)	0.310
LA-TEF (%)	45.6 (24.5–62.8)	62.0 (56.9–68.4)	65.6 (60.8–70.3)	0.055
LA-PEF (%)	27.1 (17.1–50.0)	37.4 (32.4–43.8)	37.3 (33.3–44.0)	0.413
LA-AEF (%)	12.8 (8.9–29.3)*,**	40.0 (36.0–46.0)	42.1 (38.1–49.0)	0.002
LV-GRS (%)	27.4 (21.1–32.3)	31.0 (28.4–35.5)	32.0 (28.9–35.5)	0.102
LV-GCS (%)	−17.3 (−19.2 to −14.2)	−19.4 (−21.2 to −17.8)	−18.6 (−20.7 to −17.6)	0.075
LV-GLS (%)	−14.9 ± 3.2*,**	−17.8 ± 2.3	−17.7 ± 1.4	0.019
LA reservoir strain	14.5 (8.1–24.2)*,**	38.3 (35.6–42.3)**	46.7 (42.6–50.3)	<0.001*
LA conduit strain	11.9 (3.1–15.7)*,**	24.8 (20.8–28.9)**	26.8 (24.8–28.8)	<0.001*
LA booster strain	5.2 (3.7–8.6)*,**	15.1 (11.5–16.0)**	18.4 (15.8–22.8)	<0.001*
FT3 (pmol/L)	10.4 (4.3–32.1)	12.5 (6.4–19.9)	–	0.875
TT3 (nmol/L)	4.2 (2.1–6.6)	4.1(2.2–5.2)	–	0.903
FT4 (pmol/L)	38.8 (13.2–79.0)	40.8 (16.8–63.5)	–	0.958
TT4(nmol/L)	44.6 ± 33.9	42.4 ± 27.7	–	0.865
TSH (μIU/ml)	0.05 (0.01–0.23)	0.08 (0.05–0.15)	–	0.296
BNP (pg/ml)	142.1 (62.9–413.8)	68.2 (29.2–146.6)	–	0.118

Data are the mean ± SD, percentage (number of participants), or median (interquartile range), as appropriate.

*LVEDV*, left ventricular end-diastolic volume; *LVESV*, left ventricular end-systolic volume; *LVSV*, left ventricular stroke volume; *LVEF*, left ventricular ejection fraction; *LVM*, left ventricular mass; *GRS*, global radial strain; *GCS*, global circumferential strain; *GLS*, global longitudinal strain; *LA*, left atrial; *TEF*, total emptying fraction; *PEF*, passive emptying fraction; *AEF*, active emptying fraction; *BNP*, type B natriuretic; *FT3*, serum free triiodothyronine; *TT3*, total triiodothyronine; *FT4*, free tetraiodothyronine; *TT4*, total tetraiodothyronine; *TSH*, thyroid-stimulating hormone.

*p < 0.05 (compared with the non-AF group); **p < 0.05 (compared with the control group).

Compared with the healthy controls, the hyperthyroidism patients without AF showed significantly decreased LA reservoir [38.3% (35.6–42.3) *vs*. 46.7% (42.6–50.3), *p* < 0.001], LA conduit [24.8% (20.8–28.9) *vs*. 26.8% (24.8–28.8), *p* < 0.001], and LA booster strains [15.1% (11.5–16.0) *vs*. 18.4% (15.8–22.8), *p* < 0.001]. However, the LV global myocardial strain [GLS, −17.8% ± 2.3% *vs*. −17.7% ± 1.4%, *p* = 0.818; GCS, −19.4% (−21.2 to −17.8) *vs*. −18.6% (−20.7 to −17.6), *p* = 0.208; and GRS, 31.0% (28.4–35.5) *vs*. 32.0% (28.9–35.5), *p* = 0.431] showed no significant differences between the two groups.

### Comparisons between the non-AF and AF groups

Compared with the non-AF group, the hyperthyroidism patients with AF showed a larger LVESV index (41.4 ± 6.6 *vs*. 30.6 ± 7.7 ml/m^2^, *p* = 0.005) and lower emptying fractions [LVEF, 56.2% ± 5.0% *vs*. 62.0% ± 3.9%, *p* = 0.005; LA-AEF, 12.8% (8.9–29.3) *vs*. 40.0% (36.0–46.0), *p* < 0.001].

The LA strain analysis revealed significant differences between the hyperthyroidism patients with AF and those without AF in the LA reservoir [14.5% (8.1–24.2) *vs*. 38.3% (35.6–42.3), *p* < 0.001], LA conduit [11.9% (3.1–15.7) *vs*. 24.8% (20.8–28.9), *p* < 0.001], and LA booster strains [5.2% (3.7–8.6) *vs*. 15.1% (11.5–16.0), *p* < 0.001]. In addition, the LV-GLS was significantly reduced (−14.9% ± 3.2% *vs*. −17.8% ± 2.3%, *p* = 0.016) in patients with AF compared with the non-AF group.

### Correlations between LA strain and total emptying fractions

**Figure 2:**
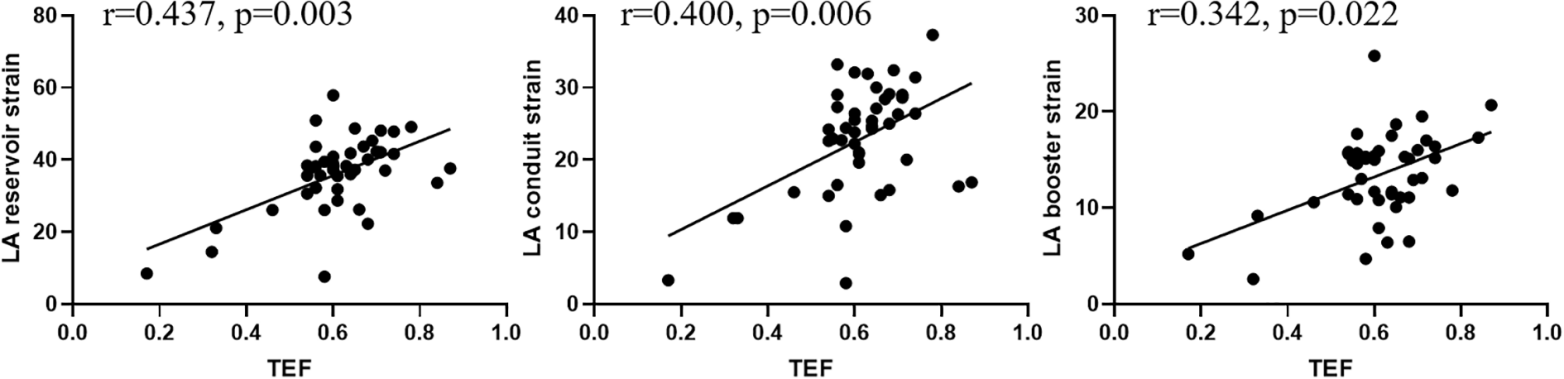
Correlations of the left atrial (LA) total emptying fraction (TEF) with the LA reservoir, conduit, and booster strains in all patients with hyperthyroidism.

LA-TEF showed significant correlations with the LA reservoir (*r* = 0.437, *p* = 0.003), conduit (*r* = 0.400, *p* = 0.006), and booster strains (*r* = 0.342, *p* = 0.022) in patients with hyperthyroidism (**Figure 2**).

### Differentiation of patients with hyperthyroidism from normal controls

**Figure 3:**
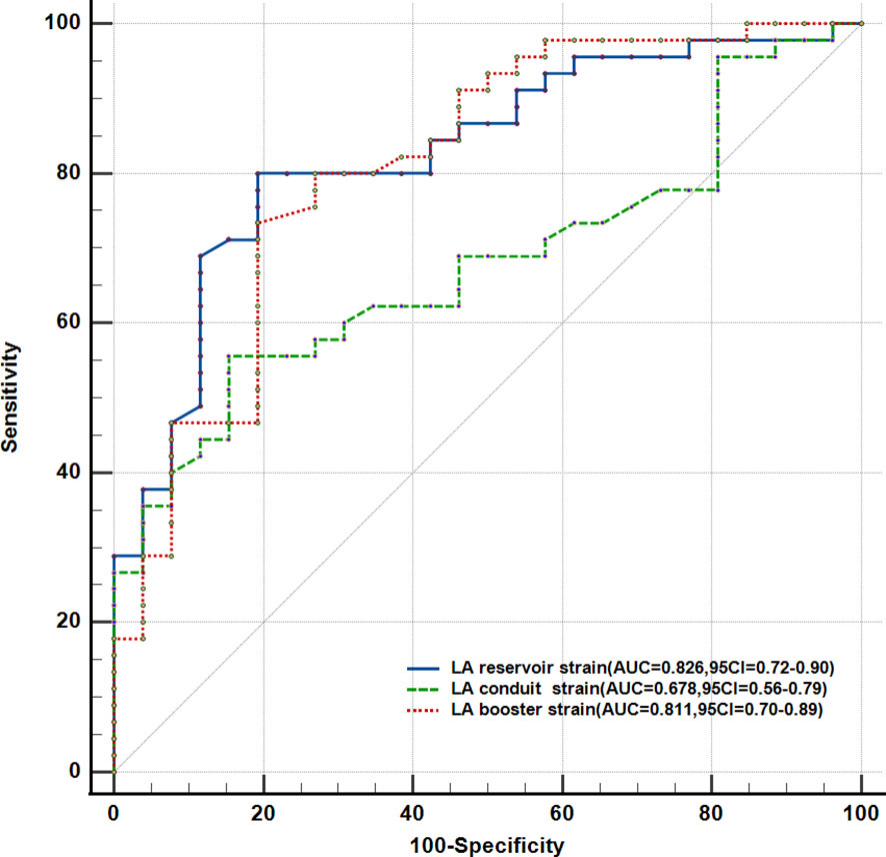
Receiver operating characteristic curve analysis of the parameters with the highest areas under the curve (AUCs).

**Figure 3** shows the ROC curves for the LA reservoir strain (cutoff value ≤42.4%, AUC = 0.826, SE = 0.050, *p* < 0.001), the LA reservoir strain (cutoff value ≤24.6%, AUC = 0.678, SE = 0.063, *p* = 0.012), and the LA booster strain (cutoff value ≤15.8%, AUC = 0.811, SE = 0.055, *p* < 0.001). The LA reservoir strain exhibited the highest AUC, which was significantly higher than that of the LA conduit strain (*p* < 0.05). Using a cutoff of 42.4% for the LA reservoir strain, patients with hyperthyroidism could be distinguished from healthy controls, with a sensitivity of 80.8% and a specificity of 80%.

### Intra- and inter-observer reproducibility

The intra-class correlation coefficients (ICCs) were 0.890, 0.880, 0.952, 0.967, 0.892, and 0.944 for the LA reservoir strain, LA conduit strain, LA booster strain, GRS, GCS, and GLS, respectively. The inter-class correlation coefficients from the interobserver analysis were 0.876, 0.883, 0.906, 0.941, 0.883, and 0.890 for the LA reservoir strain, LA conduit strain, LA booster strain, GRS, GCS, and GLS, respectively.

## Discussion

To the best of our knowledge, this is the first study evaluating both LA and LV myocardial strain using CMR-FT in patients with hyperthyroidism. This work investigated patients with hyperthyroidism and found evidence of cardiac involvement in these patients based on cardiac biomarkers. Hyperthyroidism with or without AF exhibited significantly increased heart rate and LVEDV compared with the controls. The LA-AEF and LVEF were significantly decreased in hyperthyroidism patients with AF compared with the hyperthyroidism cases without AF, although no significant differences were found in the LA-TEF, PEF, and LVMi. In the strain analyses, the LA reservoir, conduit, and booster strains were decreased in both hyperthyroidism cases with and without AF. Only the LV-GLS was decreased in the hyperthyroidism cases with AF compared with the non-AF group. The results demonstrated that the LA reservoir strain could reliably differentiate between hyperthyroidism cases with early cardiac involvement and healthy controls when the volumetric parameters and the LV strain failed to do so.

In a retrospective cross-sectional study assessing 725 patients with hyperthyroidism, the prevalence of AF was 13.8%, which corroborates this study (23). A previous study revealed LA structural and functional remodeling as a common underlying alteration in patients with AF (24). Therefore, the enrolled patients with hyperthyroidism were categorized into two subgroups, including the AF and non-AF groups.

The hyperdynamic circulation associated with hyperthyroidism, characterized by increased preload, resting heart rate, blood volume, and LV contractility, markedly increases the cardiac output (25). In this study, patients with hyperthyroidism exhibited significantly increased heart rate, LVEDV, and LVSV compared with the controls, which corroborates the current literature. The LA reservoir, conduit, and booster strains significantly decreased in patients without AF compared with healthy controls, although no significant differences in the LVEF and LA emptying fractions (TEF, PEF, and AEF) were observed. This is most likely because abnormalities in the myocardial strain do not implicate global functional impairment in the early phase.

The LA strain has been widely used to evaluate LV diastolic function in different cardiomyopathies (26–28). A previous study demonstrated that cardiovascular diseases compromising the pressure and volume overload or arrhythmic insults can result in atrial interstitial fibrosis and decrease atrial elasticity, which can be accurately detected using the LA strain (29). As shown above, the LA reservoir, conduit, and booster strains were significantly decreased prior to global systolic dysfunction in patients with hyperthyroidism. A previous work indicated that the LA strain causes a gradual progression of diastolic dysfunction, as a sensitive diagnostic tool to differentiate the stages of diastolic dysfunction (30). The LA reservoir strain (also referred to as the peak atrial longitudinal strain, PALS) may reflect the amount of LA interstitial fibrosis and is considered an important factor due to its prognostic value in cardiovascular diseases (31). In this study, the LA reservoir strain was the most accurate predictor of cardiac involvement. Our results indicate that early LV diastolic dysfunction can be detected by the LA strain in patients with hyperthyroidism. This may be explained by the nature of this parameter and the corresponding phase of the cardiac cycle depending on both the atrial mechanics itself and the mitral annular plane excursion, therefore showing a high sensitivity in the detection of diastolic dysfunction (32).

In a population-based cohort study by Olsen et al., the LA reservoir strain was associated with paroxysmal AF, independently of the LV-GLS and LA size (33). The LA reservoir strain is a marker of LA fibrosis, constituting an underlying substrate predisposing for AF, while the atrial contraction function is a marker of atrial stunning. Therefore, hyperthyroidism patients with AF had reduced LA reservoir, conduit, and booster strains compared with their counterparts without AF in this study. Incident AF in hyperthyroidism cases may also contribute to the development of LV dysfunction due to the loss of atrial contribution to LVEDV and the tachycardia-related effects on cardiac performance (34). The above results corroborate these finding as the LVEF and LV-GLS were significantly decreased only in hyperthyroidism patients with AF. A previous study demonstrated that GLS is caused by subendocardial fibers and could predict cardiovascular mortality and morbidity in the low-risk general population (14, 35). Overall, evaluation of the LA strain may help detect subtle cardiac diastolic dysfunction and explore different pathophysiological pathways, which could help guide therapeutic decisions in the future. Identifying early atrial dysfunction can help recognize hyperthyroidism patients with early cardiac involvement and provide evidence-based medicine for early medical therapy. Clinical management includes the use of anti-thyroid drugs and β-adrenoceptor blockade. Diastolic dysfunction in hyperthyroid patients who are asymptomatic for cardiac disease should be treated with anti-thyroid drugs and β-adrenoceptor blockade. Subsequently, after the thyroid function is controlled, early cardiac involvement reversibility is also assessed by measuring LA deformation.

### Limitations

The current study has several limitations. Firstly, this is a single-center retrospective study with a relatively small sample size. However, the sex ratio, age, and BMI were equally distributed between the hyperthyroidism patients and healthy controls in this research. Secondly, some of the enrolled hyperthyroidism patients had short-term treatment with anti-thyroid drugs during hospitalization. However, we ensured that all of the enrolled patients with hyperthyroidism were in a hyperthyroid state as reflected by increased T3 and T4 levels and decreased TSH levels. Thirdly, the myocardial strain was not assessed in patients with euthyroid state after treatment in this study. Whether the impaired LA myocardial stain in these patients is reversible requires further investigation. Fourthly, only 5 (11%) patients had AF in the present study, and a larger sample of hyperthyroidism patients with AF will be assessed to examine the correlation between LA strain and AF in patients with hyperthyroidism in a follow-up study by our team. However, as this is the first study to assess myocardial deformation using CMR-FT in patients diagnosed with hyperthyroidism, we believe that these findings might help better detect subtle cardiac dysfunction in patients with hyperthyroidism in actual clinical practice. Finally, a comparison of the inter-study reproducibility between the STE- and CMR-derived LA deformation parameters would have been performed due to the very thin LA wall. However, a previous study indicated that a CMR-FT-derived strain qualifies as the most reproducible parameter for LA functional assessment (19).

## Conclusion

Analysis of the LA and LV myocardial strain using CMR-FT revealed that the LA myocardial strain could reliably differentiate early cardiac involvement between hyperthyroidism patients and healthy controls, whereas volumetric parameters and ventricular myocardial strain failed to do so. These results suggest that evaluation of the LA myocardial strain should be an integral part of the diagnostic process in hyperthyroidism patients with questionable cardiac involvement, which may help detect subtle cardiac dysfunction.

## Data Availability

The original contributions presented in the study are included in the article/supplementary material. Further inquiries can be directed to the corresponding authors.
